# Protocol for differentiation of human embryonic stem cells into surface epithelium and functional keratinocytes

**DOI:** 10.1016/j.xpro.2025.103919

**Published:** 2025-06-27

**Authors:** Jiafeng Liu, Huaxing Huang, Hong Ouyang

**Affiliations:** 1 State Key Laboratory of Ophthalmology, Zhongshan Ophthalmic Center, Sun Yat-sen University, Guangdong Provincial Key Laboratory of Ophthalmology and Visual Science, Guangzhou 510060, China; 2 Center for Stem Cell Biology and Tissue Engineering, Key Laboratory for Stem Cells and Tissue Engineering, Ministry of Education, Zhongshan School of Medicine, Sun Yat-Sen University, Guangzhou 510060, China

**Keywords:** Cell Biology, Cell culture, Stem Cells

## Abstract

The normal development and function of the epidermis and other ectodermal appendages depend on the proper formation of the surface epithelium (SE). Here, we present a protocol to differentiate human embryonic stem cells (hESCs) into SE and subsequently into epidermal keratinocyte progenitors. We then detail procedures for the passage and expansion of keratinocyte progenitors, followed by 2D validation of terminal differentiation and 3D assessment of stratification potential. This protocol represents a promising advancement for stem cell-based regenerative medicine.

For complete details on the use and execution of this protocol, please refer to Liu et al.^1^ and Huang et al.^2^

## Highlights

• Protocol for differentiating surface epithelium from human embryonic stem cells
• Differentiation steps from surface epithelium to keratinocyte progenitors
• Calcium-mediated terminal differentiation in SE-derived keratinocytes
• Air-liquid interface culture system recapitulates keratinocyte stratification

## Abstract (editor highlights)

Publisher’s note: Undertaking any experimental protocol requires adherence to local institutional guidelines for laboratory safety and ethics.

## Graphical abstract

**Figure:**
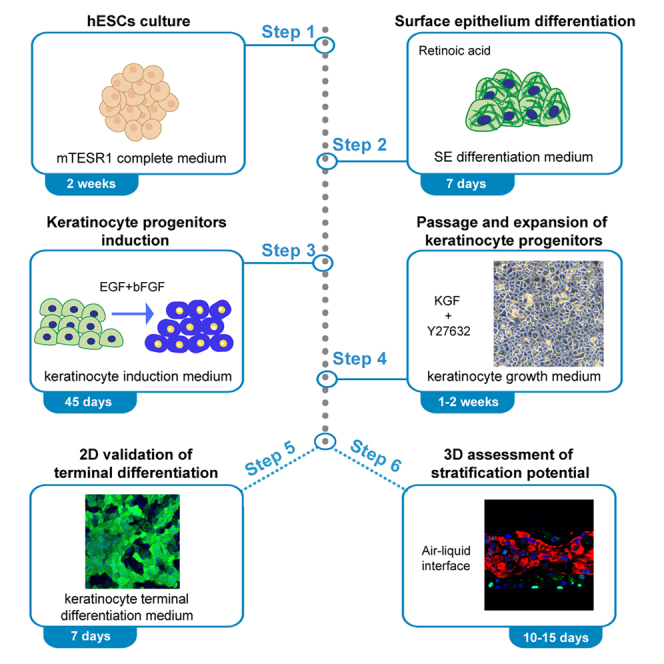

## Before you begin

This protocol provides detailed reagents and step-by-step instructions for a sequential differentiation process, mainly involving the differentiation of SE from hESCs, the generation of keratinocytes from SE, and the induction of terminally differentiated keratinocytes. While the hESC line H1 served as the primary cell model in this study, the protocol has been successfully validated for the hESC line H9 and induced pluripotent stem cell (iPSC) lines. For cell culture, hESCs were maintained in 6-well plates while differentiation was conducted in 12-well plates. All cell cultures were maintained in a humidified incubator at 37°C with 5% CO_2_. Strict sterile techniques are required throughout all procedures, and only sterile culture materials should be used to prevent potential contamination. Prior to use, hESCs and iPSCs must be verified to maintain normal karyotype and be mycoplasma-free after thawing. Additionally, cultured and differentiated cells should undergo routine mycoplasma testing every two months. For qRT-PCR analysis, biological replicates (n=3) were generated by three independent rounds of cell culture and differentiation using the same cell line, with each replicate processed separately.

### Institutional permissions

The human pluripotent stem cells used in this study are approved for research use by the Ethics Committee of Zhongshan Ophthalmic Center. Researchers who wish to replicate this protocol will need approval from their respective institutions.

### Diluting hESC-qualified matrix and coating plates

**Timing: 1 day**

hESCs will be cultured and differentiated on tissue culture-treated polystyrene plates coated with Corning Matrigel hESC-qualified Matrix. Prior to dilution, confirm the dilution factor based on the specific lot number of the hESC-qualified Matrix.

1. Thaw the hESC-qualified Matrix at 4°C for 24 h.
2. Pre-chill 1.5 mL microcentrifuge tubes and pipette tips to 4°C.
3. Aliquot the hESC-qualified Matrix into pre-chilled 1.5 mL microcentrifuge tubes according to the dilution factor.
4. Dilute one aliquot of the hESC-qualified Matrix in 25 mL of cold DMEM/F-12 medium and mix thoroughly. Store the remaining aliquots at −80°C for up to 6 months for future use.

**CRITICAL:** Handle the Corning Matrigel hESC-qualified Matrix on ice to prevent premature gel polymerization. Aliquoted hESC-qualified Matrix should avoid repeated freezing and thawing, and the diluted Matrix should be used promptly to ensure optimal performance.

5. Coat the plates with the diluted hESC-qualified Matrix at a volume of 1 mL/well for 6-well plates or 0.5 mL/well for 12-well plates.
6. Incubate the coated plates at 37°C for at least 60 min.

**CRITICAL:** Ensure complete coverage of the well surfaces during coating to achieve uniform cell culture conditions, avoid prolonged incubation of coated plates at 37°C for more than 48 h to prevent drying out. Coated culture plates can be stored at 4°C for up to one week (Parafilm-sealed to prevent matrix drying out and potential contamination).

### Diluting type I collagen and coating plates

**Timing: 70 min**

The induced epidermal keratinocyte progenitors will be passaged, cultured and differentiated on tissue culture-treated polystyrene plates coated with type I collagen.

7. Pre-chill 50 mL centrifuge tubes and pipette tips to 4°C.
8. Dilute type I collagen in ice-cold PBS at a 1:49 dilution ratio using the pre-chilled 50 mL centrifuge tubes and mix thoroughly.
9. Coat the plates with the diluted type I collagen solution, using 1 mL/well for 6-well plates or 0.5 mL/well for 12-well plates, ensuring complete coverage of the well surfaces.
10. Incubate the coated plates at 37°C for a minimum of 60 min. The incubation period at 37°C should not exceed 48 h to avoid matrix drying out. Unused coated plates can be stored at 4°C for up to one week when properly sealed with Parafilm to prevent drying out and contamination.

***Note:*** Type I collagen dilution must be conducted under cold conditions to prevent premature gelation.

### Reconstituting and aliquoting reagents

**Timing: 1 h**

This section details procedures for reagent preparation, including reconstitution, aliquoting, and storage of compounds required for culture and differentiation media. Briefly centrifuge lyophilized powders to ensure complete collection. Aliquot the stock solutions into single-use volumes. Avoid repeated freeze-thaw cycles.

11. Dissolve retinoic acid (RA) powder in DMSO to prepare a 20 mM stock solution. Aliquot the RA stock solution and store at −80°C. Keep a single working aliquot at −20°C for up to 1 month.

**CRITICAL:** RA is light-sensitive. All procedures including dissolution, usage, and storage should be performed under light-protected conditions to ensure compound stability.

12. Dissolve Epidermal Growth Factor (EGF) and basic Fibroblast Growth Factor (bFGF) powders separately in sterile distilled water to prepare 100 μg/mL stock solutions.

**CRITICAL:** Maintain EGF and bFGF stock solutions at ≥50 μg/mL concentration to ensure stability.

13. Prepare stock solutions by dissolving Keratinocyte Growth Factor (KGF) and ROCK inhibitor Y27632 powders in sterile distilled water. The final stock concentrations should be 50 μg/mL for KGF and 100 mM for Y27632, respectively.

### Preparing culture media and differentiation media

**Timing: 1 h**

14. Preparation of hESC culture medium.
  a. Thaw the 5× supplement from the mTeSR1 complete kit at 4°C for 24 h.
  b. Add the thawed supplement to the mTeSR1 basal medium and mix thoroughly.
  c. Aliquot the prepared mTeSR1 complete medium and store at −80°C for up to 6 months. Maintain a single working aliquot at 4°C for up to 2 weeks.
15. Preparation of SE differentiation medium.
  a. Add RA to mTeSR1 complete medium to a final concentration of 1 μM.
  b. Store the prepared SE differentiation medium in the dark at 4°C for up to 2 weeks.

***Note:*** The SE differentiation medium should be stored under light-protected conditions to ensure compound stability.

16. Preparation of keratinocyte induction medium.
  a. Thaw the supplement of Defined Keratinocyte-SFM (DKSFM) at 15°C–25°C.
  b. Add the thawed supplement to DKSFM basal medium and mix thoroughly.
  c. Add EGF and bFGF to the DKSFM medium to a final concentration of 10 ng/mL each.
  d. Mix thoroughly and store the keratinocyte induction medium in the dark at 4°C.

***Note:*** Protect DKSFM components (basal medium, supplement) and prepared medium from light exposure.

17. Preparation of keratinocyte growth medium.
  a. Add the prepared stock solutions to the complete DKSFM medium to achieve final working concentrations of 10 ng/mL for KGF and 10 μM for Y27632.
  b. Mix thoroughly and store the prepared keratinocyte growth medium in the dark at 4°C.
18. Preparation of keratinocyte terminal differentiation medium.
  a. Add 1 M CaCl_2_ solution to the keratinocyte growth medium to a final concentration of 1.2 mM.
  b. Mix thoroughly. Store the keratinocyte terminal differentiation medium in the dark at 4°C.

### Thawing and culturing hESCs

**Timing: 2 h**

19. Pre-warm the mTeSR1 complete medium and DMEM/F-12 medium to 15°C–25°C.
20. Coat a 6-well tissue culture-treated polystyrene plate with Corning Matrigel hESC-qualified Matrix (refer to the “diluting hESC-qualified Matrix and coating plates” section for detailed instructions).
21. Retrieve the frozen hESC line from the liquid nitrogen; place the cryovial in a 37°C water bath. Gently swirl the vial for about 3 min, until only a small ice crystal remains (approximately 90% thawed).

***Note:*** Gently agitating the cryovial in the 37°C water bath ensures uniform heating and speeds up the thawing process.

22. Transfer the cell cryopreservation solution to a 50 mL centrifuge tube, and slowly add 10 mL pre-warmed DMEM/F-12 medium.

***Note:*** Avoid pipetting up and down to prevent dissociation of cell colonies.

23. Centrifuge the cells at 280 *g* for 5 min at 25°C. Aspirate the supernatant without disturbing the cell pellet.
24. Add 1 mL pre-warmed mTeSR1 complete medium to resuspend the cells.

**CRITICAL:** Pipette up and down with a P1000 pipette only once to resuspend the cells and prevent excessive dissociation of colonies.

25. Aspirate the hESC-qualified Matrix from the coated 6-well plate. Add 2 mL/well pre-warmed mTeSR1 complete medium to the plate.

***Note:*** Perform this step during the cell centrifugation process.

26. Seed the resuspended cell suspension into the 6-well plate at a 1:15 to 1:20 passage ratio to achieve a density of approximately 300–400 colonies/cm^2^.

***Note:*** The passage ratio is determined by the cryopreserved cell count. A 1:15 to 1:20 ratio represents the cell quantity obtained from one well of a 6-well plate at 60%–70% confluency prior to freezing. Adjust ratio based on the cryopreserved cell count.

27. Gently shake the plate to ensure even distribution of the colonies, and incubate the plate at 37°C in a 5% CO_2_ tissue culture incubator.
28. Replace the mTeSR1 complete medium daily. Passage the hESCs every 4–5 days upon reaching 50%–60% confluence.

**CRITICAL:** If suboptimal cell attachment is observed at 24-h post-thawing, supplement the culture medium with 10 μM Y27632 during the initial 24-h recovery period to enhance cell survival and promote colony formation.

## Key resources table

**Table:** 

REAGENT or RESOURCE	SOURCE	IDENTIFIER
**Antibodies**		

Mouse monoclonal anti-KRT18, dilution 1:500	Invitrogen	Cat#MA5-12104
Rabbit monoclonal anti-TP63, dilution 1:200	Cell Signaling Technology	Cat#67825
Mouse monoclonal anti-KRT14, dilution 1:500	Invitrogen	Cat#MA5-11599
Mouse monoclonal anti-KRT1, dilution 1:500	Santa Cruz	Cat#SC-376224
Mouse monoclonal anti-KRT10, dilution 1:500	Invitrogen	Cat#MA1-06319
Anti-rabbit, Alexa Fluor 488, dilution 1:1,000	Thermo Fisher Scientific	Cat#A11008
Anti-mouse, Alexa Fluor 488, dilution 1:1,000	Thermo Fisher Scientific	Cat#A11001
Anti-rabbit, Alexa Fluor 594, dilution 1:1,000	Cell Signaling Technology	Cat#8889S
Anti-mouse, Alexa Fluor 594, dilution 1:1,000	Cell Signaling Technology	Cat#8890S

**Chemicals, peptides, and recombinant proteins**		

DMEM/F12	Gibco	Cat#C11330500BT
mTeSR1	STEMCELL Technologies	Cat#85850
Defined Keratinocyte-SFM	Thermo Fisher Scientific	Cat#10744019
Matrigel hESC-qualified matrix	Corning	Cat#354277
Type I collagen	Sigma	Cat#C3867
Trypsin-EDTA (0.25%)	Thermo Fisher Scientific	Cat#25200072
Defined trypsin inhibitor	Thermo Fisher Scientific	Cat#R007100
Retinoic acid (RA)	R&D Systems	Cat#0695
Epidermal growth factor (EGF)	Millipore	Cat#GF144
Basic fibroblast growth factor (bFGF)	MedChemExpress	Cat#HY-P7004
Keratinocyte growth factor (KGF)	MedChemExpress	Cat#HY-P70673
ROCK inhibitor Y27632	Tocris	Cat#1254
Calcium chloride (CaCl_2_) solution	Sigma	Cat#21115
Gentle Cell Dissociation Reagent	STEMCELL Technologies	Cat#07174
Phosphate-buffered saline (PBS)	Thermo Fisher Scientific	Cat#C10010500BT
Dimethylsulfoxide (DMSO)	Sigma	Cat#D2650
Formalin solution, neutral buffered, 10%	Sigma	Cat#HT501128
Ethyl alcohol	Sigma	Cat#E7023
DNase/RNase-free distilled water	Thermo Fisher Scientific	Cat#10977015
DAPI	Sigma	Cat#D9542

**Experimental models: Cell lines**		

hESCs line H1	Laboratory of Nan Cao	N/A
hESCs line H9	Laboratory of Nan Cao	N/A

**Oligonucleotides**		

NANOG-FW: TTTGTGGGCCTGAAGAAAACT	This paper	N/A
NANOG-RV: AGGGCTGTCCTGAATAAGCAG	This paper	N/A
POU5F1-FW: CTGGGTTGATCCTCGGACCT	This paper	N/A
POU5F1-RV: CCATCGGAGTTGCTCTCCA	This paper	N/A
SOX2-FW: CTCGTGCAGTTCTACTCGTCG	This paper	N/A
SOX2-RV: AGCTCTCGGTCAGGTCCTTT	This paper	N/A
KRT7-FW: TCCGCGAGGTCACCATTAAC	This paper	N/A
KRT7-RV: GCTCTGTCAACTCCGTCTCAT	This paper	N/A
KRT8-FW: GATCGCCACCTACAGGAAGCT	This paper	N/A
KRT8-RV: ACTCATGTTCTGCATCCCAGACT	This paper	N/A
KRT18-FW: CCGTCTTGCTGCTGATGACT	This paper	N/A
KRT18-RV: GGCCTTTTACTTCCTCTTCGTG	This paper	N/A
TP63-FW: TTTCCCACCCCGAGATGA	This paper	N/A
TP63-RV: TGCGGCGAGCATCCAT	This paper	N/A
KRT5-FW: CCAAGGTTGATGCACTGATGG	This paper	N/A
KRT5-RV: TGTCAGAGACATGCGTCTGC	This paper	N/A
KRT14-FW: TGCCGAGGAATGGTTCTTCACC	This paper	N/A
KRT14-RV: GCAGCTCAATCTCCAGGTTCTG	This paper	N/A
ITGB4-FW: CTGTACCCGTATTGCGACT	This paper	N/A
ITGB4-RV: AGGCCATAGCAGACCTCGTA	This paper	N/A
LAMB3-FW: GCAGCCTCACAACTACTACAG	This paper	N/A
LAMB3-RV: CCAGGTCTTACCGAAGTCTGA	This paper	N/A
COL7A1-FW: ACCAGCAATGGCTATGCTAAAA	This paper	N/A
COL7A1-RV: GCCTCGTGTGCTTCCAGTT	This paper	N/A
KRT1-FW: CAGCATCATTGCTGAGGTCAAGG	This paper	N/A
KRT1-RV: CATGTCTGCCAGCAGTGATCTG	This paper	N/A
KRT10-FW: TCCTACTTGGACAAAGTTCGGG	This paper	N/A
KRT10-RV: CCCCTGATGTGAGTTGCCA	This paper	N/A
FLG-FW: TGAAGCCTATGACACCACTGA	This paper	N/A
FLG-RV: TCCCCTACGCTTTCTTGTCCT	This paper	N/A
LOR-FW: GTCTGCGGAGGTGGTTCCTCT	This paper	N/A
LOR-RV: TGCTGGGTCTGGTGGCAGATC	This paper	N/A
TGM1-FW: GCACCACACAGACGAGTATGA	This paper	N/A
TGM1-RV: GGTGATGCGATCAGAGGATTC	This paper	N/A

**Other**		

6-well tissue culture-treated plates	Corning	Cat#3516
12-well tissue culture-treated plates	Corning	Cat#3513
24-well tissue culture-treated plates	Corning	Cat#3526
1.5 mL microcentrifuge tubes	Corning	Cat#MCT-150-C
15 mL centrifuge tubes	Corning	Cat#430791
50 mL centrifuge tubes	Corning	Cat#430829
6.5 mm Transwell with 0.4 μm pore polyester membrane insert	Corning	Cat#3470
Forma 3111 tissue culture incubator	Thermo Fisher Scientific	N/A
Water bath	Thermo Fisher Scientific	Cat#18007A-1CEQ
Hemocytometer	Marienfeld	N/A
DMiL Inverted phase contrast microscope	Leica	N/A
DMi8 Inverted fluorescence microscope	Leica	N/A
QuanStudio 7 FLex Real-Time PCR System	Thermo Fisher Scientific	Cat#4485701

## Step-by-step method details

### Seeding hESCs for SE differentiation

**Timing: 2 h**

This section describes the steps for seeding hESCs maintained in 6-well plates at 50%–60% confluence, one day prior to initiating SE differentiation. For optimal cell conditions, hESCs should undergo 1–2 routine passages post-thaw before the pre-differentiation passage.

1. Pre-warm the mTeSR1 complete medium and DMEM/F-12 medium to 15°C–25°C.

***Note:*** Avoid warming of the medium in a 37°C water bath. Pre-warm the medium at 15°C–25°C.

2. Coat 12-well tissue culture-treated plates with Corning Matrigel hESC-qualified Matrix (refer to the “before you begin” section for detailed instructions).
3. Aspirate the medium from the 6-well plate containing hESCs.
4. Gently wash the hESCs with 1 mL DMEM/F-12 medium and aspirate the wash solution.
5. Add 1 mL Gentle Cell Dissociation Reagent to the hESC colonies and incubate at 37°C in culture incubator for 3 min. Aspirate the Gentle Cell Dissociation Reagent after incubation.

***Note:*** The required volume of reagents depends on the culture vessel. For instance, the volumes of DMEM/F-12 medium and Gentle Cell Dissociation Reagent described herein are standardized for 6-well plates, whereas half volumes are recommended for 12-well plate applications.

6. Add 1 mL pre-warmed mTeSR1 medium to the hESC colonies.
7. Scrape the hESC colonies gently using a cell scraper until most cells are detached from the plate.
8. Gently pipette the cell suspension up and down 3–4 times with a P1000 pipette.

**CRITICAL:** The size of hESC colonies is crucial for achieving high SE differentiation efficiency. Do not over- or under-pipetting to maintain appropriately sized colonies. Formation of single-cell suspensions should be avoided.

9. Aspirate the hESC-qualified Matrix from the coated 12-well plates.
10. Add 1 mL pre-warmed mTeSR1 medium to each well of the coated 12-well plates.
11. Seed the resuspended cell suspension into the 12-well plate at a 1:30 passage ratio to achieve a density of approximately 100–200 colonies/cm^2^.

**CRITICAL:** The density of hESC colonies is critical for achieving high differentiation efficiency.

12. Gently shake the plate to ensure even distribution of the colonies.
13. Incubate the plate at 37°C in a 5% CO_2_ tissue culture incubator for 24 h.

### Directed differentiation of hESCs into SE

**Timing: 7 days**

This section describes the stepwise differentiation of hESCs into SE. The initial density and size of hESC colonies are critical factors for ensuring successful differentiation. Prior to initiating differentiation, the cell confluence should be approximately 10%–20%, and the colonies should ideally be 100–200 μm in diameter for optimal differentiation efficiency.

14. Pre-warm the SE differentiation medium to 15°C–25°C before use.

***Note:*** Avoid warming of the medium in a 37°C water bath. Pre-warm the medium at 15°C–25°C to prevent degradation of RA and other medium components.

**Figure 1:**
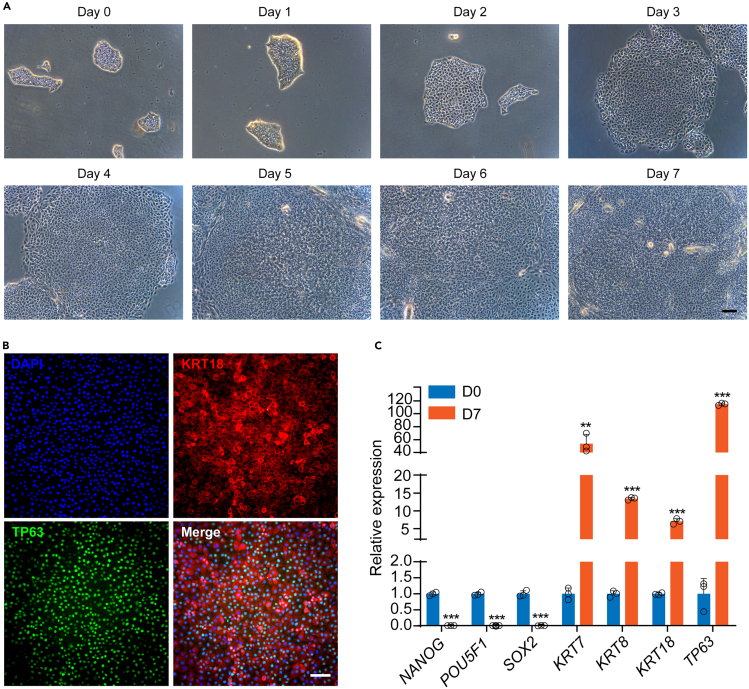
Directed differentiation of hESCs into SE (A) Phase contrast images showing the differentiating hESCs during 7 days of RA induction. Scale bar: 100 μm. (B) Immunostaining for KRT18 and TP63 in the differentiated cells on D7. Scale bar: 100 μm. (C) qRT-PCR analysis of representative genes in hESCs and cells after seven days of differentiation. qRT-PCR values were normalized to the values in hESC group. Values are presented as means ± SD (n=3 biological replicates; ∗∗P<0.01; ∗∗∗P<0.001, t test).

15. Aspirate the medium from the 12-well plate containing pre-seeded hESCs.
16. Add 1 mL/well of pre-warmed SE differentiation medium.
17. Maintain at 37°C, 5% CO_2_ with daily monitoring of colony morphological transitions (Figure 1A).
18. Replace the SE differentiation medium every two days (48 h).
19. Harvest the differentiated cells on day 7 to assess differentiation efficiency. Perform both immunostaining and quantitative PCR (qPCR) analysis of SE markers (KRT7, KRT8, KRT18, and TP63), using 2–3 wells from a 12-well plate for each assay (Figures 1B and 1C). Following verification of successful SE differentiation (≥80% KRT18^+^/TP63^+^ cell population), proceed with keratinocyte progenitor differentiation using the remaining cells.

### Directed differentiation of SE into keratinocyte progenitors

**Timing: 45 days**

This section describes the steps for differentiating mature SE cells into keratinocyte progenitors. The differentiation efficiency of SE cells is crucial for the successful induction of keratinocyte progenitors.

**Figure 2:**
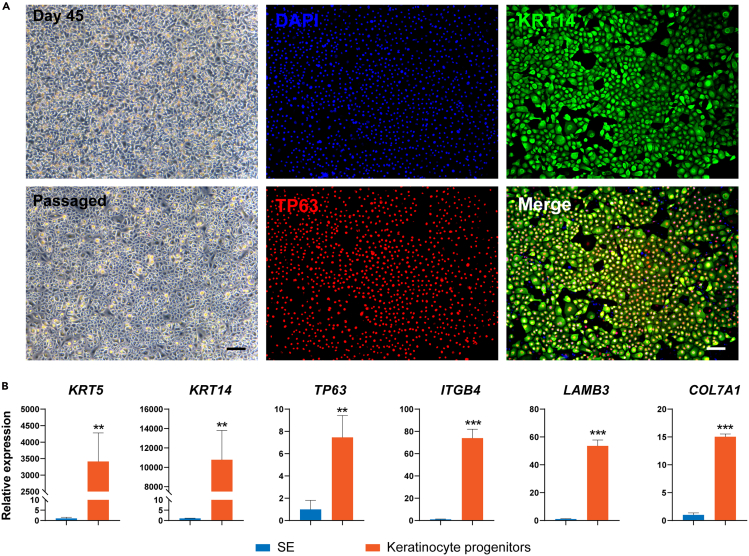
Induction of SE cells into mature keratinocyte progenitors (A) Left panel: Phase contrast images of the induced keratinocyte progenitors after 45 days of differentiation and 4 days after passaging. Scale bar: 100 μm. Right panel: Immunostaining for KRT14 and TP63 in the induced keratinocyte progenitors. Scale bar: 100 μm. (B) qRT-PCR analysis of representative genes in SE and induced keratinocyte progenitors. qRT-PCR values were normalized to the SE group. Values are presented as means ± SD (n=3 biological replicates; ∗∗P<0.01; ∗∗∗P<0.001, t test).

20. Pre-warm the keratinocyte induction medium to 15°C–25°C before use.
21. Aspirate SE differentiation medium completely from 12-well plates.
22. Add 1 mL/well pre-warmed keratinocyte induction medium.
23. Maintain the plate at 37°C in a humidified tissue culture incubator with 5% CO_2_.
24. Replace the keratinocyte induction medium every day (24 h).
25. Maintain the cells in culture for 45 days to achieve the induction of mature keratinocyte progenitors. Assess progenitor markers via immunofluorescence and qPCR analysis at day 45 of differentiation (Figure 2).

***Note:*** During the SE-to-keratinocyte progenitor transition, limited cell turnover (balanced death and proliferation) represents normal differentiation dynamics. However, excessive cell death (<40% confluency) or epithelial-mesenchymal transition (EMT; characterized by fibroblast-like morphology) typically indicates incomplete SE commitment, suboptimal cell health or contamination. These aberrant events will significantly compromise differentiation efficiency, resulting in failed keratinocyte progenitor generation**.**

### Passage and expansion of keratinocyte progenitors

**Timing: 2 h**

This section describes the steps for subculturing and expanding 45-day differentiated keratinocyte progenitors. The keratinocyte progenitors are expanded and cultured on tissue culture-treated 12-well plates coated with type I collagen.

26. Coat 12-well plates with type I collagen (refer to the “before you begin” section for detailed instructions).
27. Pre-warm the keratinocyte growth medium to 15°C–25°C before use.
28. Pre-warm trypsin-EDTA (0.25%) in a 37°C water bath for at least 15 min.
29. Aspirate the keratinocyte induction medium from the 12-well plate containing keratinocyte progenitor cells.
30. Gently wash the cells twice with 1 mL of 1× PBS (without calcium and magnesium) per well. Aspirate the wash solution after each wash.
31. Add 500 μL of 37°C trypsin-EDTA (0.25%) to each well and incubate the plate at 37°C in a tissue culture incubator for 3–5 min, or until the cells begin to detach.
32. Gently pipette the cell suspension up and down 5–10 times to ensure complete detachment and dissociation of cells.
33. Add 500 μL of defined trypsin inhibitor (DTI) to each well to neutralize the trypsin-EDTA after incubation.

**CRITICAL:** Add DTI after complete cell detachment is achieved.

34. Transfer the cell suspension to a 15 mL centrifuge tube.
35. Centrifuge the cells at 280 *g* for 5 min at 25°C.
36. Carefully aspirate the supernatant without disturbing the cell pellet.
37. Add 1 mL pre-warmed keratinocyte growth medium and resuspend the cell pellet using a P1000 pipette with gentle pipetting.
38. Count the number of cells using either a hemocytometer or automated cell counter.
39. Aspirate the type I collagen from the coated 12-well plates. Add 1 mL of pre-warmed keratinocyte growth medium to each well.
40. Seed 5 × 10^4^ cells into each well of the 12-well plate. Gently shake the plate in a cross-shaped motion to ensure even distribution of cells.
41. Maintain the plate at 37°C in a 5% CO_2_ tissue culture incubator.
42. Monitor cell confluency daily. Passage or cryopreserve the cells when they reach 90%–95% confluency (Figure 2A).

### Induction of terminal differentiation in keratinocyte progenitors

**Timing: 7 days**

This section describes the calcium-induced terminal differentiation protocol for expanded keratinocyte progenitors, with terminal differentiation progression assessed through specific marker expression (KRT1, KRT10, FLG, LOR and TGM1). The cells are treated with 1.2 mM CaCl_2_ in the differentiation medium for 7 days at full confluence.

43. Pre-warm the keratinocyte terminal differentiation medium to 15°C–25°C before use.

**CRITICAL:** Ensure accurate CaCl_2_ concentration (1.2 mM final) through proper preparation of stock and working solutions.

**Figure 3:**
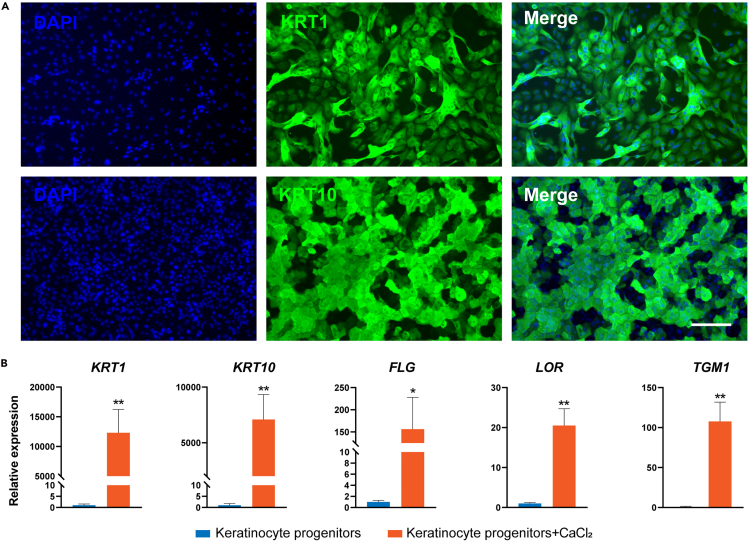
Terminal differentiation of keratinocyte progenitors (A) Immunostaining for KRT1 and KRT10 in the keratinocytes after 7 days of CaCl_2_ treatment. Scale bar: 100 μm. (B) qRT-PCR analysis of representative genes in keratinocyte progenitors and keratinocytes after 7 days of CaCl_2_ treatment. qRT-PCR values were normalized to the keratinocyte progenitor group. Values are presented as means ± SD (n=3 biological replicates; ∗P<0.05; ∗∗P<0.01, t test).

44. Aspirate the keratinocyte growth medium from confluent cultures.
45. Add 1 mL pre-warmed keratinocyte terminal differentiation medium to each well.
46. Incubate the plate at 37°C in a humidified tissue culture incubator with 5% CO_2_.
47. Replace the keratinocyte terminal differentiation medium every day (24 h).
48. Culture the cells for 7 days to achieve terminal differentiation of keratinocyte progenitors. Assess differentiation efficiency via immunofluorescence and qPCR analysis (Figure 3).

### Air-lifting culture assay of keratinocyte progenitors

**Timing: 10–15 days**

**Figure 4:**
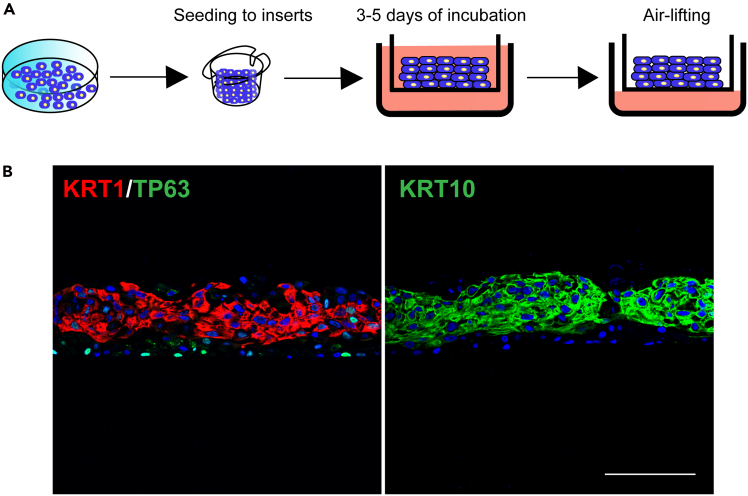
Air-lifting assay of keratinocyte progenitors (A) Schematic diagram illustrating the procedure of the air-lifting culture assay. (B) Immunostaining for KRT1, KRT10 and TP63 in the stratified keratinocytes after 10 days of air-lifting induction. Scale bar: 100 μm.

This section describes the steps for culturing keratinocyte progenitors in an air-liquid interface system to validate their stratification potential (Figure 4). Cells are cultured in type I collagen-coated transwell inserts (0.4-μm polyester membrane) within a 24-well culture plate. Following seeding in transwell, the cells formed a multilayered structure. Upon air-liquid interface induction, they organized into differentiated suprabasal layers (KRT1/KRT10^+^) and a TP63^+^ stem cell-retaining basal layer, faithfully recapitulating native epidermal organization (Figure 4).

49. Coat the transwell inserts of 24-well culture plate with 200 μL of type I collagen (refer to the “before you begin” section for detailed instructions).
50. Pre-warm the keratinocyte induction medium to 15°C–25°C before use.
51. Pre-warm trypsin-EDTA (0.25%) in a 37°C water bath for at least 15 min.
52. Aspirate the keratinocyte growth medium from the 12-well plate containing expanded keratinocyte progenitor cells.
53. Gently wash the cells twice with 1 mL 1× PBS (without calcium and magnesium) per well. Aspirate the wash solution after each wash.
54. Add 500 μL 37°C trypsin-EDTA (0.25%) to each well and incubate the plate at 37°C in a tissue culture incubator for 5 min.
55. Gently pipette the cell suspension up and down 5–10 times using a P1000 pipette to ensure complete detachment and dissociation of cells into single cell suspension.
56. Add 500 μL of DTI to each well to neutralize the trypsin-EDTA.
57. Transfer the cell suspension to a 15 mL centrifuge tube and centrifuge at 280 *g* for 5 min at 25°C.
58. Carefully aspirate the supernatant without disturbing the cell pellet.
59. Resuspend the cell pellet in 1 mL of pre-warmed keratinocyte induction medium by gently pipetting.
60. Count the number of cells using a hemocytometer or automated cell counter.
61. Aspirate the type I collagen from the coated transwell inserts.
62. Seed 7×10^5^ cells into each transwell insert and add keratinocyte induction medium to a final volume of 200 μL in the inserts.
63. Add 500 μL of keratinocyte induction medium to the lower chambers of the 24-well plate.
64. Maintain the plate at 37°C in a humidified tissue culture incubator with 5% CO_2_ for 3–5 days. Replace the medium in both the upper inserts and lower chambers every day.

***Note:*** A critical 3–5 day incubation period following cell seeding into transwell inserts is required prior to air-lifting initiation, ensuring (1) complete cellular attachment and spreading, (2) proper intercellular junction formation, and (3) microenvironmental adaptation.

65. Carefully aspirate the medium from the upper inserts and adjust the volume of medium in the lower chamber to 200 μL to raise the cells to an air-liquid interface.
66. Maintain the cells at the air-liquid interface for 7–10 days to mimic the stratification process of keratinocyte progenitors.
67. Fix the samples with 10% neutral buffered formalin, followed by dehydration, embedding, sectioning, and immunofluorescence staining (Figure 4).

## Expected outcomes

The formation of the SE represents a fundamental aspect of vertebrate embryonic development, serving as the precursor for multiple vital structures, particularly the epidermis.^3^ Here, we present a comprehensive protocol for the directed differentiation of hESCs into SE lineages, characterized by robust expression of definitive SE markers including KRT7, KRT8/18, and TP63^4^ (Figure 1). Throughout the pluripotent-to-SE transition, cells undergo characteristic morphological changes: initial colony flattening and emergence of epithelial-like cells (days 1–3), followed by establishment of a uniform cobblestone morphology (days 4–7) that reflects stable SE commitment. Through subsequent induction, these SE cells progressively differentiate into epidermal keratinocyte progenitors displaying characteristic epithelial morphology and expressing definitive progenitor markers KRT5, KRT14, TP63, ITGB4, LAMB3 and COL7A1^5^ (Figure 2). This differentiation platform not only enables detailed investigation of SE and epidermal developmental mechanisms but also facilitates the identification of key transcriptional regulators governing these processes. Importantly, the derived keratinocyte progenitors establish a physiologically relevant human *in vitro* model that recapitulates key aspects of epidermal biology, enabling investigations of cutaneous pathophysiology and drug evaluation, as demonstrated through comprehensive functional validation of the progenitor population. Terminal differentiation assays confirm their maturation capacity, with calcium induction generating keratinocytes expressing differentiation markers KRT1, KRT10, FLG, LOR, and TGM1^6^ (Figure 3). Moreover, air-liquid interface culture reveals their stratification competence, forming organized basal (TP63^+^) and suprabasal (KRT1^+^, KRT10^+^) layers that mimic native epidermal architecture (Figure 4).

## Limitations

This protocol enables the generation of highly pure SE cells and keratinocyte progenitors through optimized differentiation procedures. However, the differentiation efficiency of SE is critically dependent on the clonal size and seeding density of hESCs, which may exhibit line-to-line variability across different hESC sources. Notably, insufficient size/density can predispose cells toward mesenchymal lineage differentiation, whereas excessive clonal expansion promotes neural lineage commitment. Therefore, systematic optimization of these parameters is essential when encountering suboptimal differentiation outcomes, requiring empirical determination of the ideal clonal characteristics for each specific hESC line to ensure reproducible and efficient SE differentiation. Furthermore, the current protocol requires an extended 45-day induction period for the differentiation of SE cells into keratinocyte progenitors, representing a significant temporal limitation that warrants optimization in future studies to enhance the efficiency and practicality of this differentiation platform. Although the induced keratinocytes generated by our differentiation protocol demonstrate cellular morphologies, marker gene expression profiles, and differentiation potential closely resembling human primary epidermal stem cells, our transcriptomic analysis revealed significantly reduced expression of immune-related genes (e.g., *GBP2*, *GBP3*, *RIPK2*, *IRAK4*, and *ANXA3*) associated with inflammatory pathways. We hypothesize that this difference may stem from the lack of physiologically relevant microenvironmental cues in the *in vitro* culture system, a limitation that warrants systematic investigation in future studies.

## Troubleshooting

### Problem 1

Thawed hESCs exhibit suboptimal growth characteristics, manifested by low post-thaw viability, aberrant cellular morphology and increased spontaneous differentiation (before you begin step 28).

### Potential solution

• Avoid using long-term cryopreserved cells. Do not thaw hESCs that have been stored in liquid nitrogen for extended periods (>2 years).
• Minimize mechanical stress during cell suspension pipetting, both during thawing and passaging procedures.
• Implement regular mycoplasma testing (e.g., monthly). Immediately discard contaminated cultures and establish new cultures from mycoplasma-free stocks.

### Problem 2

Poor SE differentiation efficiency is observed, characterized by the inability of cells to adopt epithelial-like morphology and a low proportion of KRT18^+^ and TP63^+^ cells (<50%) by Day 7 (step-by-step method details step 19).

### Potential solution

• Optimize seeding density and clonal size. Different cell lines exhibit variable growth rates and response to small-molecule stimuli differently. Seed cells at a density of approximately 100–200 colonies/cm^2^, ensuring colonies remain non-confluent during early differentiation (Day 1–4). By late differentiation (Day 7), colonies should form a near-confluent monolayer without overcrowding.
• Use fresh SE differentiation medium. RA is light-sensitive and prone to degradation. Prepare and store RA-containing medium in dark conditions. Avoid prolonged heating in a 37°C water bath. Do not store prepared medium at 4°C for extended periods (>2 week).
• Confirm the stock and working concentrations of RA to ensure proper differentiation.

### Problem 3

Excessive cell death during keratinocyte progenitor induction (step-by-step method details step 25).

### Potential solution

• Ensure successful SE lineage commitment. Inefficient SE differentiation directly compromises subsequent keratinocyte progenitor induction. Verify successful SE differentiation by confirming ≥80% KRT18^+^ and TP63^+^ cell population via immunofluorescence staining before proceeding.
• Maintain medium integrity. Keratinocyte induction medium components are light- and temperature-sensitive. Prepare fresh medium weekly, avoid heating in a water bath, and pre-warm at 20°C–25°C.

### Problem 4

Low post-passage attachment efficiency of keratinocyte progenitors (step-by-step method details step 42).

### Potential solution

• Avoid prolonged trypsin-EDTA digestion and minimize vigorous pipetting of cell suspensions.
• Coat plates with properly diluted type I collagen (stored at 4°C to prevent premature gelation). Ensure sufficient collagen is added to each well to prevent drying.
• Optimize seeding density to achieve 90%–95% confluency within approximately 4 days of culture.

### Problem 5

The stratified keratinocyte layers cultured in the air-lifting system exhibit insufficient thickness and lack appropriate markers in both the basal and suprabasal layers (step-by-step method details step 67).

### Potential solution

• Increase seeding density. Adjust the cell number to 1–1.2 × 10^6^ cells per transwell insert.
• Extend pre-air-lift incubation. Maintain submerged culture conditions for 5 days post-seeding before transitioning to the air-liquid interface.
• Minimize mechanical disruption. Exercise caution during medium changes to avoid disturbing the cells in the transwell inserts.

## Resource availability

### Lead contact

Further information and requests for resources and reagents should be directed to and will be fulfilled by the lead contact, Hong Ouyang (ouyhong3@mail.sysu.edu.cn).

### Technical contact

Technical inquiries regarding this protocol should be directed to and will be answered by the technical contact, Jiafeng Liu (liujf7@mail2.sysu.edu.cn).

### Materials availability

Materials associated with this protocol are available upon request from the lead contact.

### Data and code availability

This protocol does not report datasets or generate code.

## Acknowledgments

This work was funded by the 10.13039/100014717National Natural Science Foundation of China (no. 82271043), the 10.13039/100014717National Natural Youth Science Foundation of China (no. 32400597), the 10.13039/501100018540Natural Science Foundation of Guangdong Province (no. 2023A1515012719), and the 10.13039/501100002858China Postdoctoral Science Foundation (no. 2023M734023). We would like to thank Professor Nan Cao for sharing H1 and H9 human embryonic stem cells.

## Author contributions

H.O. supervised the project and reviewed the manuscript. J.L. and H.H. performed the experiments and wrote the manuscript.

## Declaration of interests

The authors declare no competing interests.
